# An Explainable Fuzzy Framework for Assessing Preeclampsia Classification

**DOI:** 10.3390/biomedicines13061483

**Published:** 2025-06-16

**Authors:** Matías Salinas, Daira Velandia, Leondry Mayeta-Revilla, Ayleen Bertini, Marvin Querales, Fabian Pardo, Rodrigo Salas

**Affiliations:** 1 PhD Program in Health Sciences and Engineering, Universidad de Valparaíso, Valparaíso 2540064, Chile; leondry.mayeta@postgrado.uv.cl (L.M.-R.); ayleen.bertini@uv.cl (A.B.); 2 Biomedical Engineering School, Faculty of Engineering, Universidad de Valparaíso, Valparaíso 2362905, Chile; 3 Center of Interdisciplinary Biomedical and Engineering Research for Health—MEDING, Universidad de Valparaíso, Valparaíso 2540064, Chile; marvin.querales@uv.cl; 4 Millennium Institute for Intelligent Healthcare Engineering (iHealth), Santiago 7820436, Chile; 5 Metabolic Diseases Research Laboratory (MDRL), School of Medicine, Faculty of Medicine, San Felipe Campus, Universidad de Valparaíso, San Felipe 2172972, Chile; 6 Statistical Institute, Faculty of Science, Universidad de Valparaíso, Valparaíso 2360102, Chile; daira.velandia@uv.cl; 7 Medical Technology School, Faculty of Medicine, Universidad de Valparaíso, Valparaíso 2540064, Chile

**Keywords:** disorders in pregnancy, preeclampsia, fuzzy systems, explainable machine learning

## Abstract

**Background:** Preeclampsia remains a leading cause of maternal morbidity worldwide. There is a critical need for predictive systems that not only perform accurately but also provide interpretable insights for clinical decision-making. This work introduces SK-MOEFS, an explainable framework based on fuzzy logic and multi-objective evolutionary optimization, designed to classify preeclampsia risk while generating clinically interpretable rules. **Methods:** The model integrates fuzzy decision trees with a genetic algorithm to identify a compact and relevant set of rules, optimized for both accuracy and interpretability. The system was trained and evaluated on third-trimester pregnancy data from a publicly available, multi-ethnic cohort comprising 574 individuals. All processes, including preprocessing, training, and evaluation, were conducted using open-source tools, ensuring reproducibility. **Results:** SK-MOEFS achieved 91% classification accuracy, an AUC of 0.89, and a recall of 0.88—outperforming other standard interpretable models while maintaining high transparency. The model emphasizes minimizing false negatives, which is critical in clinical risk stratification for preeclampsia. **Conclusions:** Beyond predictive performance, SK-MOEFS offers a rule translation and defuzzification layer that outputs probabilistic interpretations in natural language, enhancing its suitability for clinical use. This framework provides an effective bridge between algorithmic inference and human clinical judgment, supporting transparent and reliable decision-making in maternal care.

## 1. Introduction

Approximately 10% of pregnancies worldwide are complicated by hypertensive disorders, and about 10% to 15% of maternal deaths are due to preeclampsia/eclampsia, which are common phenotypic manifestations of hypertension [1]. Preeclampsia is a pregnancy syndrome characterized by arterial hypertension onset after 20 gestational weeks, with or without proteinuria, associated with other organic alterations (platelets, liver dysfunction, and kidney failure, among others), with an incidence estimate between 2 and 8% among pregnant women [2,3]. The prevalence of preeclampsia has reached 5.6% in Chile [4].

Recent studies have explored the application of artificial intelligence (AI) to predict preeclampsia, demonstrating varying levels of predictive performance depending on the algorithms and clinical data employed. Marić et al. [5] implemented an elastic-net regularized regression model with data obtained before 16 weeks of gestation, achieving an AUC of 0.79 for early prediction. Jhee et al. [6] applied a stochastic gradient boosting model to a longitudinal dataset from the early second trimester up to 34 weeks, reporting an AUC of 0.924 for late-onset preeclampsia. Similarly, Liu et al. [7] and Zhang et al. [8] employed ensemble models like random forest and LightGBM, respectively obtained during first-trimester screening, achieving AUCs of 0.86 and 0.89. Despite these advances, conventional ML algorithms rely heavily on manually engineered features and often lack the ability to convey predictions through explicit interpretable representations of clinical knowledge [9].

Recent developments in deep learning have introduced hybrid models that incorporate fuzzy logic to enhance both predictive accuracy and interpretability—key aspects for clinical applications such as preeclampsia screening [10,11,12,13]. Although originally applied to non-medical datasets, these architectures can be adapted to clinical contexts by encoding domain-specific thresholds (e.g., blood pressure and age) into fuzzy rules, supporting transparent and semi-supervised decision-making processes [14,15,16].

Across the studies, several core variables consistently emerged as predictive inputs, including maternal demographic data (age, BMI, and parity), medical and obstetric history, vital signs (blood pressure), and a range of laboratory markers and medication use. These variables were primarily captured during routine prenatal care in the first trimester or in the context of high-risk monitoring in later stages.

In clinical applications, model reliability encompasses not only predictive accuracy but also interpretability—crucial for fostering trust among healthcare providers and patients [17]. In high-stakes environments such as obstetric care, the use of opaque black-box models can hinder clinical adoption. Explainable artificial intelligence (XAI) frameworks have therefore gained relevance, offering strategies to elucidate model behavior and enhance decision-making [18]. Within this context, fuzzy inference systems based on fuzzy logic have proven effective in translating complex data into interpretable fuzzy logic rules, enabling multi-level reasoning that aligns with clinical thinking [19].

Research has increasingly focused on explainable artificial intelligence (XAI) methods to enhance the interpretability of predictive models for preeclampsia, with the intent of improving transparency and facilitating clinical adoption. For instance, Nugroho et al. [20] developed a fuzzy-logic-based expert system that incorporates 51 if–then rules to model clinical reasoning under conditions of uncertainty. This system employs Mamdani inference to produce interpretable outputs. Likewise, Espinilla et al. [19] proposed a mobile monitoring system that integrates fuzzy linguistic modeling with decision trees (C4.5 algorithm). This system applies a linguistic transformation to ten clinical variables, resulting in a rule-based tree consisting of 197 nodes, which aids in the early detection of high-risk pregnancies. However, the use of rules may complicate interpretation as the number of rules increases [13,21]. A recent contribution by Pham et al. [22] introduced fuzzy knowledge graphs (FKGs), which automatically generate rule-like structures by combining symptomatic attributes with physician preferences and clinical data to enhance diagnostic support. Nonetheless, this method does not fully disclose the internal decision-making processes, thus limiting transparency in situations where clinical justification is essential. While these methodologies exhibit significant potential for developing interpretable and clinically aligned AI systems, certain limitations persist. These challenges underscore the necessity for balanced approaches that maintain both predictive utility and semantic clarity within medical decision-making.

Fuzzy-rule-based systems (FRBSs) have emerged to effectively provide explainability to data-driven models using linguistic rules that humans can interpret. One example is multi-objective evolutionary algorithms (MOEAs), which optimize problems with multiple conflicting objectives by evolving a population of candidate solutions [23]. MOEFSs (multi-objective evolutionary fuzzy systems) leverage the strengths of both FRBSs and MOEAs, enabling the creation of transparent and efficient models. These algorithms have been applied in various healthcare areas, such as disease prediction, treatment optimization, and patient risk assessment [24,25,26,27,28].

This research aims to present a novel fuzzy framework for classifying preeclampsia patients based on a multi-objective evolutionary fuzzy system (MOEFS) algorithm. Our approach leverages this algorithm to derive various fuzzy partitions, enhancing the explainability of predictions. This methodology preserves prediction performance and significantly improves model interpretability, potentially boosting end-user confidence in clinical settings. The proposed framework offers an approach that connects data-driven inference with clinical reasoning. Using fuzzy rules, membership functions, and defuzzification metrics, it enables transparent result interpretation and allows clinicians to refine outputs based on their expertise.

The key technical contributions of this study include (i) the use of a fuzzy logic system within a multi-objective model (SK-MOEFS), which allows balanced predictive accuracy with clinical relevance; (ii) the automatic generation of interpretable rules for clinical decision-making; (iii) a focus on clinical interpretability through the use of visual rule outputs and linguistic variable mappings; and (iv) the construction of a multi-ethnic clinical dataset to support model training and evaluation in diverse populations.

The paper is organized as follows. In Section 2, we highlight the significance of FRBS models and formalize the underlying principles. Section 3 describes the proposed framework, developed using a multi-objective evolutionary algorithm (MOEA) approach. In Section 4, we present a simulation study demonstrating the framework’s performance in preeclampsia prediction and its ability to generate interpretable outputs. Section 5 reflects on the findings and outlines the key challenges for future research. Finally, the conclusion summarizes the main contributions and their clinical relevance.

In summary, the proposed method not only classifies preeclampsia risk but also logically explains the assumptions on which this classification is based, providing clinicians with interpretable insights into key maternal risk factors.

## 2. Theoretical Framework

### 2.1. Types of Explanations

In the context of machine learning applied to clinical decision-making, the interpretability of models plays a critical role in ensuring transparency, trust, and ethical compliance [29,30]. This section classifies the types of explanations into two main dimensions: scope and methodological dependency.

First, explanations can be categorized as *global*—those that provide insight into the model’s overall behavior—or *local*—those that elucidate individual predictions [31]. In clinical applications, both are essential: global explanations aid in understanding general patterns, while local explanations support case-specific interpretability, particularly in complex diagnostic scenarios such as preeclampsia risk prediction.

Second, explanation techniques can be *model-agnostic* or *model-specific*. Model-agnostic methods, such as SHAPs (SHapley Additive exPlanations) [32] and LIMEs (Local Interpretable Model-Agnostic Explanations) [33], are applicable to any black-box model, offering post hoc interpretability. In contrast, model-specific techniques are designed to work only with particular model structures.

Among them, SHAPs stand out due to their foundation in game theory and additive feature attributions, formalized as

(1) $$g\left(z^{\prime }\right)=\phi_{0}+\sum_{j=1}^{M}\phi_{j}z_{j}^{\prime }$$

where $g(z^{\prime })$ is the interpretable approximation, $\phi_{j}$ represents the contribution of feature *j*, and $z_{j}^{\prime }$ indicates the presence of a feature in the simplified input vector. This mathematical representation enables consistent and locally accurate explanations.

Additionally, the section distinguishes between *ante hoc* and *post hoc* interpretability. Ante hoc models, such as fuzzy-rule-based systems, are inherently transparent, requiring no additional explanation mechanisms after training [34]. Post hoc approaches, conversely, rely on surrogate models or perturbation-based methods to approximate the reasoning behind complex models. By establishing this taxonomy, the section underscores the rationale for adopting fuzzy logic systems in clinical settings, where native interpretability and linguistic reasoning offer a compelling advantage over opaque predictive architectures [35].

### 2.2. Fuzzy-Rule-Based Systems

An FRBS comprises two main components: the Knowledge Base (KB) and the fuzzy inference engine. The KB consists of linguistic rules and parameters defining fuzzy sets used in these rules [36]. The fuzzy inference engine then utilizes this information to predict outcomes when new input patterns are introduced.

Let $X=\{X_{1},\ldots ,X_{F}\}$ denote the set of input attributes and *Y* the output attribute. Let $U_{f}$, with f=1,…,F+1, represent the universe of the $f^{th}$ attribute $X_{f}$. Let $P_{f}=\left\{A_{f,1},\ldots ,A_{f,T_{f}}\right\}$ be a fuzzy partition of $T_{f}$ fuzzy sets on attribute $X_{f}$. A frequently used type of fuzzy partition is the triangular partition $A_{f,j}$. Thus, each fuzzy set $A_{f,j}$ can be described by its membership function $MF(A_{f,j})$, represented as a tuple $\left(a_{f,j},b_{f,j},c_{f,j}\right)$. Here, $a_{f,j}$ and $c_{f,j}$ denote the left and right boundaries of the support of $A_{f,j}$, while $b_{f,j}$ represents its core. A triangular strong fuzzy partition uses overlapping triangular membership functions, where the peak of one triangle coincides with the boundary (base) of its neighboring triangles, ensuring a smooth transition between sets. The training dataset $\left\{\left(x_{1},y_{1}\right),\ldots ,\left(x_{N},y_{N}\right)\right\}$ is defined as a collection of *N* input–output pairs.

In classification scenarios, *Y* is categorical, with the *t*-th sample $y_{t}\in C$, where $C=\{C_{1},\ldots ,C_{K}\}$ denotes the set of *K* possible classes. To ascertain the class of a given input vector, one can employ a fuzzy-rule-based classifier (FRBC) with a rule-based (RB) structure composed of *M* rules, expressed as follows:

$$\begin{array}{cc} R_{m}: & \mathrm{IF}X_{1}\mathrm{is}A_{1,j_{m,1}}\mathrm{AND}\ldots \mathrm{AND}X_{f}\mathrm{is}A_{f,j_{m,f}}\mathrm{AND}\ldots \mathrm{AND}X_{F}\mathrm{is}A_{F,j_{m,F}}\\ \; & \mathrm{THEN}Y\mathrm{is}C_{k}\mathrm{with}RW_{m} \end{array}$$

where $C_{k}$ denotes the labels associated with the $m^{th}$ rule, and $RW_{m}$ is the rule weight of each element in C. Consequently, the FRBC provides an estimated output value or class label, utilizing a specific inference engine that depends on the strength of activation of each rule with the input.

### 2.3. Multi-Objective Evolutionary Algorithms (MOEAs)

Multi-objective evolutionary algorithms (MOEAs) are optimization techniques designed to simultaneously address multiple conflicting objectives, such as classification accuracy and model interpretability—both essential in clinical decision support systems. The Vector-Evaluated Genetic Algorithm (VEGA) was the first MOEA proposed [37], introducing the concept of Pareto optimality to manage trade-offs between objectives. In this context, MOEAs have been integrated with fuzzy-rule-based systems (FRBSs) to create genetic fuzzy systems (GFSs), where evolutionary processes are employed to generate and refine fuzzy rules [38,39].

**Figure 1:**
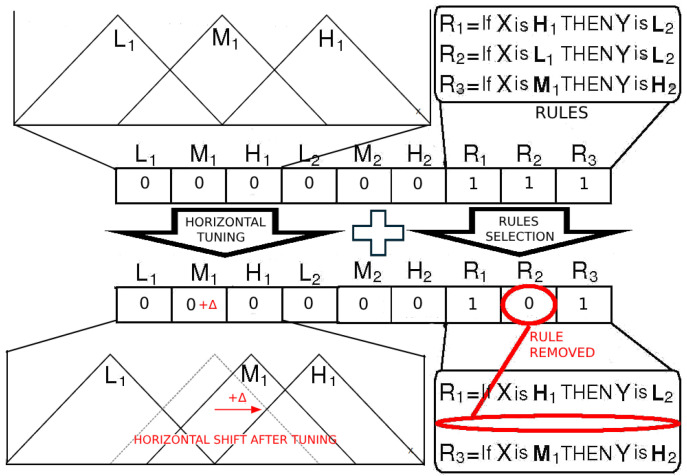
Example of genetic Algorithm Tuning: In this example, a candidate membership function was generated by applying a horizontal shift to the original. This represents one of several types of modifications that the genetic algorithm can perform, such as deleting or splitting membership functions. The red circle indicates a rule that was removed during the selection process. The red circle highlights a rule that was removed during the selection process. Adapted from [40].

Specifically, rule selection via genetic algorithms allows the elimination of redundant, conflicting, or non-contributory rules, thereby improving both performance and comprehensibility. The Michigan approach is adopted, wherein each chromosome represents a single fuzzy rule [38]. This facilitates evolutionary competition at the rule level and leads to the selection of concise semantically relevant rules (Figure 1). Genetic operators such as mutation and crossover are applied to enhance diversity and promote convergence to optimal solutions. Mutation operators introduce small random changes to chromosomes, enhancing diversity and allowing the exploration of new areas in the search space. Crossover operators combine parts of two parent chromosomes to create new offspring, promoting the inheritance of good traits from both parents.

The hybridization of fuzzy systems and genetic algorithms has given rise to GFSs, which offer several advantages for machine learning despite not being initially designed for this purpose [41].

To quantify interpretability, the total rule length (TRL) metric is used, measuring the complexity of a ruleset [38]. Candidate solutions are then categorized as first (high accuracy, lower interpretability), last (low accuracy, high interpretability), or median (balanced), allowing flexible deployment depending on clinical context. This evolutionary strategy provides a principled framework for constructing fuzzy systems that are both effective and explainable.

## 3. Materials and Methods

**Figure 2:**
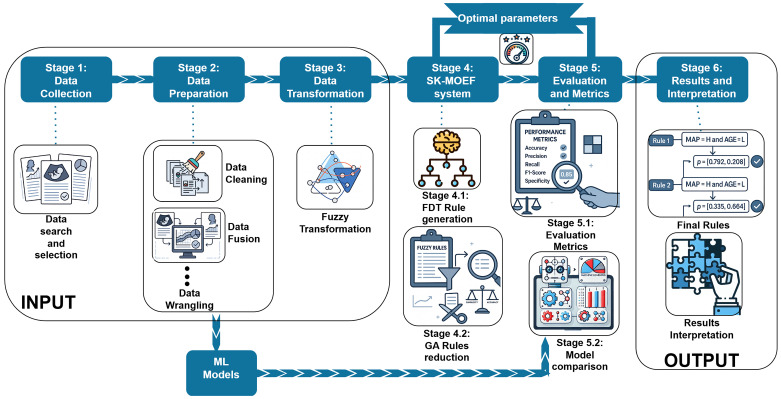
Proposed framework. The first stage covers data collection, followed by data preparation. The next step involves data transformation, applying fuzzification techniques and decision-tree-based rule knowledge. For data mining, we utilize the SK-MOEFS algorithm to select the best model and reduce the number of rules. Next is the evaluation phase, which employs performance metrics to assess the results. Finally, the presentation of results summarizes the defuzzification process and the final outputs of the classification.

The methodological framework of this study is structured to ensure transparency and clinical relevance in preeclampsia risk prediction. As summarized in Figure 2, the explainable fuzzy framework integrates the complete modeling process, from data collection and preparation, through fuzzification, to evaluation and interpretation of results. The following subsections describe each component in detail, including the construction of a multi-ethnic clinical dataset, the integration of fuzzy logic into a fuzzy engine, and the comparative evaluation against classical models that do not undergo the fuzzification stage.

The explainable fuzzy framework integrates the entire clinical modeling pipeline, from raw data collection to interpretable decision outputs. Clinical data is first cleaned, fused, and transformed into fuzzy linguistic terms (e.g., MAP = high; age = low), enabling the system to explicitly model clinical reasoning. These fuzzified inputs are processed through a fuzzy decision tree system, where the fuzzy logic rules are optimized using a genetic algorithm. This genetic optimization, which acts as the core mechanism for fuzzy logic refinement, balances model precision and interpretability by iteratively adjusting rule weights, pruning redundant rules, and enhancing coverage of clinically relevant cases. The final model outputs probabilistic rules and classification results aligned with domain knowledge and clinical reasoning standards. In contrast, classical models such as decision trees and random forests are applied directly to the cleaned but non-fuzzified data, serving as benchmarks for performance comparison. The framework outputs both standard evaluation metrics (e.g., TP, FP, FN, and TN) and interpretable fuzzy rules, enabling clinicians to understand how predictions are made and supporting transparent decision-making in preeclampsia risk assessment.

In what follows, we describe in more detail each stage involved in this framework.

### 3.1. Stage 1: Data Collection

The dataset used in this study was constructed by integrating four publicly available clinical databases, each providing relevant information for the classification of preeclampsia (PE). This integration aimed to build a heterogeneous and clinically grounded sample that reflects diverse patient populations. The selected datasets were obtained from prior research studies that explored maternal cardiovascular and metabolic variables during pregnancy, all of which included sufficient clinical features to enable harmonization. The datasets incorporated into the final sample were as follows: *(i)* Petry et al. [42], which analyzed the association between age at menarche and blood pressure among 438 pregnant women from the Cambridge Baby Growth Study; *(ii)* Tatapudi et al. [43], a prospective case-control study of 100 women conducted in India to assess cardiovascular function under varying severity levels of hypertensive disorders; *(iii)* Thitivichienlert et al. [44], a study with 34 PE-diagnosed patients aimed at examining the relationship between renal biomarkers and long-term blood pressure outcomes; and *(iv)* Pham et al. [22], which proposed a fuzzy-knowledge-graph-based diagnostic model trained on a cohort of 210 pregnant women, from which 198 labeled cases were usable for this work.

Across these datasets, five clinical variables were selected as common denominators to enable structured integration: mean arterial pressure (MAP), body mass index (BMI), maternal age (age), parity (number of prior births), and gestational age (GA). These variables were chosen for their recognized predictive value in PE detection and their consistent presence across all data sources. To ensure variable comparability, MAP and BMI values were recalculated using standard clinical formulas. MAP was computed from systolic (SYS) and diastolic (DIA) blood pressure readings using Equation (2):

(2) $$MAP\left(SYS,DIA\right)=\frac{1}{3}\times SYS+\frac{2}{3}\times DIA$$

BMI was calculated from the patient’s weight and height using Equation (3):

(3) $$BMI\left(height,weight\right)=\frac{weight}{height^{2}}$$

A preliminary quality assessment of each dataset was performed to ensure the adequacy of their descriptive statistics and the compatibility of their measurement units and variable definitions. Table 1 summarizes the total number of records, the class distribution (PE vs No-PE), and the descriptive statistics (mean ± standard deviation and range) for each clinical variable used in this study.

As shown in Table 1, the Tatapudi and Pasumarthy dataset included only three of the five variables: MAP, age, and parity. Meanwhile, the other datasets contained some missing data (Table 2). The mixed dataset consisted of 574 patients and showed variability in MAP and BMI ranges, reflecting the heterogeneous clinical conditions across the different cohorts. This multi-source composition enriched the dataset’s representation and diversity but also introduced a notable class imbalance (72 PE vs. 502 No-PE), which was addressed in later stages of modeling through evaluation strategies that emphasized recall and sensitivity. The methodological integration of these datasets thus established a solid foundation for subsequent preprocessing, fuzzification, and classification stages, enabling a reliable and explainable framework for PE risk assessment.

### 3.2. Stage 2: Data Preparation

This stage involved data curation procedures including variable filtering, imputation of missing values, normalization, and error correction. The goal was to prepare a unified dataset suitable for robust and interpretable classification under the explainable fuzzy framework.

Initially, retain only variables common across all sources. The selected datasets were merged by matching common variables, preserving structural integrity across entries. This fusion process resulted in an integrated dataset containing observations with partial missingness in some features, particularly in BMI, age, and GA. Table 2 summarizes the distribution of missing values across the datasets that required imputation.

To handle this incompleteness, we applied a comparative evaluation of multiple imputation methods available from the KEEL (Knowledge Extraction based on Evolutionary Learning) platform. Specifically, we applied 18 different imputation algorithms from this toolbox, including methods such as PCA, K-means, and fuzzy K-means, among others. Bayesian PCA (BPCA) was selected based on its ability to preserve the distributional properties of the data in alignment with reference values reported in the literature for third-trimester pregnant Indian population [43]. In BPCA models, the dataset *Y* is a linear combination of latent factors with Gaussian noise:

(4) Y=WZ+E

Here, *W* represents the matrix of principal component loadings, *Z* the latent variable scores, and *E* the residual error. The model parameters are estimated using an expectation–maximization (EM) procedure combined with Bayesian estimation, which provides robustness in the presence of high-dimensional and sparsely observed data [45,46]. Oba et al. [47] further discuss the differences between BPCA and other imputation methods, such as Singular Value Decomposition (SVD) and k-Nearest Neighbor (k-NN), in the context of imputation tasks.

After applying BPCA to the datasets with incomplete features, we examined the imputed values to confirm that they remained within clinically plausible ranges. Table 3 shows the summary statistics of the imputed values for the Tatapudi dataset, with stratification by PE status. The values are consistent with known distributions of BMI and GA in late pregnancy, providing confidence in the reliability of the imputation.

The statistical descriptive analysis of the results of the imputation process is shown in Table 3. According to the work of Gunton et al. [48], our mean and standard deviation align with the average values for the third-trimester pregnant Indian population.

Thus, the final dataset, whose descriptive statistics are summarized in Table 4, consisted of 574 observations with five features each.

### 3.3. Stage 3: Data Transformation

Upon completing the data preparation process, each clinical feature was encoded using strong triangular membership functions, as detailed in Section 2.2, where the formal structure and parameters $\left(a_{f,j},b_{f,j},c_{f,j}\right)$ for fuzzy sets were defined. This structure ensures full domain coverage, smooth overlap between adjacent sets, and computational efficiency [39].

Initially, a partitioning algorithm is used to create preliminary clusters, with centroids selected based on a long-distance measure. This results in a fuzzy dataset, which serves as the foundation for subsequent steps. The number of membership functions per variable was initially set uniformly and later optimized during the learning stage (see Section 3.4).

This transformation step generated a linguistically rich, structurally coherent fuzzy dataset aligned with the principles of XAI, which are critical in clinical decision-making contexts [33,49].

### 3.4. Stage 4: SK-MOEFS

At the core of this methodology is the application of scikit-learn-based multi-objective evolutionary fuzzy system (SK-MOEFS) [36]. This system integrates fuzzy logic with machine learning to generate a set of fuzzy rules that balance interpretability and accuracy.

We have applied the grid-based method to search for the optimal parameters of the SK-MOEFS model. The number of membership functions per variable was set to five. There are 10.368 possible combinations shown in Table 5, where each combination was run ten times.

#### 3.4.1. Optimal Set of Parameters for the SK-MOEFS Method

For each parameter configuration, the confusion matrix was analyzed to extract true positive (TP) and false positive (FP) rates, and these results were plotted unordered to help visualize and identify the best-performing configurations for the minority class. This criterion showed that the genetic algorithm could go from poorly predicting the minority class with the “median” TRL to effectively characterizing it with the “last” TRL. Configurations that did not meet these criteria were discarded. The focus was on those that showed improved performance for the minority class despite potentially reduced performance for the majority class.

This process reduced the original 10,368 parameter combinations to just over 100. These 100 parameter combinations were re-executed 100 times each to validate their stability. In order to further filter out these combinations, the following criteria were applied:

1. The “median” model’s confusion matrix must exhibit better performance in classifying the minority class compared to the original model from which rules were extracted.
2. The original tree model from which rules were extracted must start with at least 100 rules.
3. Combinations of parameters were selected based on the TN–FN and TP–FP normalized metrics.
4. Combinations of parameters with low density were excluded and evaluated using a histogram.
5. The “last” model should outperform traditional models in classifying the minority class.

The first two criteria ensured a minimum number of acceptable rules for the model’s multi-objective optimization. Different mechanics can be established to achieve the third criterion; however, the one with the greatest visual potential is a joinplot between the normalized TN–FN and TP–FP difference, which allows us to observe the cases where the normalized differences are maximum (more details see Section 4.1).

The fourth criterion was addressed by examining the histograms of each parameter and discarding those with low counts suggesting instability in achieving the desired result. This process resulted in a pool of five combinations. The fifth and final criterion considered both quantitative as well as arbitrary components. Combinations were compared and the one with the highest frequency of expected outcomes in the “last” model was selected while generating more than 2 final rules. This ensured that the model did not overfit to a single class and maintained a balance between the two classes.

A particular aspect of SK-MOEFS is that the membership functions for each parameter needed to be established. The model was limited to a fixed number of variable membership functions.

#### 3.4.2. Stage 4.1: Rule Generation Using Fuzzy Decision Tree

The generation of the initial fuzzy rule base in the SK-MOEFS framework is performed using a fuzzy decision tree (FDT) learning strategy. This method recursively partitions the fuzzified feature space into subsets that maximize class discrimination, allowing the extraction of linguistically interpretable **if–then** rules. The methodology follows the principles proposed by Segatori et al. [50], tailored for explainable decision-making in clinical applications.

Each internal node of the tree represents a fuzzy condition over a clinical variable (e.g., “MAP is high”), while each path from the root to a leaf defines a complete fuzzy classification rule. The selection of the splitting attribute at each node is governed by the *fuzzy information gain (FGain)*:

(5) $$FGain\left(P_{f};IG\right)=FEnt\left(G\right)-WFEnt\left(P_{f};IG\right)$$

where FEnt(G) denotes the fuzzy entropy of the current node, and $WFEnt(P_{f};IG)$ is the weighted fuzzy entropy of the partition induced by attribute *f*.

The entropy for each fuzzy subset $A_{f,j}$ is computed as

(6) $$FEnt\left(A_{f,j}\right)=\sum_{k=1}^{K}-\frac{|SC_{k}^{I_{f},j}|}{|A_{f,j}|}\log_{2}\left(\frac{|SC_{k}^{I_{f},j}|}{|A_{f,j}|}\right)$$

where $|SC_{k}^{I_{f},j}|$ is the fuzzy cardinality of samples in class $C_{k}$, and

(7) $$|A_{I_{f},j}|=\sum_{i=1}^{N_{I_{f},j}}\mu_{A_{I_{f},j}}\left(x_{i,f}\right)$$

For each child node $G_{j}$, the fuzzy cardinality is computed as

(8) $$|G_{j}|=\sum_{i=1}^{N_{j}}T_{N}\left(\mu_{A_{f,j}}\left(x_{f,i}\right),\mu_{G}\left(x_{i}\right)\right)$$

For categorical features, this simplifies to

(9) $$|G_{j}|=\sum_{i=1}^{N_{j}}T_{N}\left(1,\mu_{G}\left(x_{i}\right)\right)$$

The recursive growth of the tree continues until any of the following stopping criteria are satisfied:

- All instances in the node belong to the same class;
- The number of samples is below the threshold λ;
- The maximum tree depth β is reached;
- The fuzzy information gain is less than $\varepsilon =10^{-6}$.

When a leaf node is created, it is not assigned a crisp class label. Instead, a fuzzy class distribution is computed, representing the degree of membership of the data in the node to each class based on fuzzy cardinalities. This preserves classification uncertainty and enhances clinical interpretability.

The overall procedure is summarized in Algorithm 1.
**Algorithm 1** Fuzzy Decision Tree Construction (adapted from Segatori et al., [50]). 1:**Function** BuildFuzzyTree (dataset *G*, depth *d*) 2:**if** stopping criteria are met **then** 3:   Compute fuzzy cardinality of *G* for each class 4:   **Return** leaf node with fuzzy class distribution 5:**end if** 6:**for all** feature $X_{f}$ and fuzzy set $A_{f,j}$ **do** 7:   Compute fuzzy information gain (Equations (5)–(7)) 8:**end for** 9:Select $X_{f}^{*}$ and $A_{f,j}^{*}$ with highest gain10**for all** partitions $G_{j}$ induced by $A_{f,j}^{*}$ **do**11:   Compute fuzzy cardinality (Equation (8) or (9))12:   **Recursively call** BuildFuzzyTree($G_{j}$, d+1)13:**end for**14:**Return** decision node with branches to $G_{j}$ subtrees

Each complete path in the tree defines a fuzzy rule such as

IFMAPisHighANDBMIisMediumTHENClass=[0.7PE,0.3No-PE]

This rule format supports probabilistic reasoning and is particularly useful for uncertain or borderline clinical scenarios. The set of rules extracted in this stage constitutes the initial hypothesis space for the subsequent optimization phase (see Section 3.4.3).

#### 3.4.3. Stage 4.2: Reduce Fuzzy Rules Using Genetic Algorithm

After the complete fuzzy rule base is generated through the FDT (Section 3.4.2), a multi-objective genetic algorithm is employed to select a minimal yet effective subset of rules.

The genetic algorithm evolves this population across several generations, applying crossover and mutation operators to explore different rule combinations. The objective is to maximize the classifier’s predictive quality while minimizing rule complexity. This trade-off is managed through a fitness function that evaluates each solution based on

- Normalized classification performance, using sensitivity and specificity metrics.
- Interpretability, measured by the TRL, which penalizes lengthy rules to favor more concise readable solutions.

Throughout the evolution process, individuals are ranked using a Pareto-based sorting scheme, enabling the algorithm to maintain a diverse set of non-dominated solutions. The final fuzzy rule base is selected from the Pareto front, balancing classification accuracy and linguistic simplicity.

This stage contributes significantly to improving the interpretability and generalizability of the SK-MOEFS classifier, reducing model redundancy while preserving semantic and clinical coherence in the ruleset. The fundaments are in the previous Section 2.3.

### 3.5. Stage 5: Evaluation and Metrics

#### 3.5.1. Stage 5.1: Evaluation Metrics

The performance of the SK-MOEFS classifier and comparative models was evaluated using well-established classification metrics derived from the confusion matrix. This matrix includes four key quantities: true positive (TP), true negative (TN), false positive (FP), and false negative (FN). These values form the foundation of several indicators that assess both predictive accuracy and clinical assistive diagnostics [51,52], and are summarized in Table 6.

The primary metrics used include the following:

- Accuracy—measures the proportion of correct predictions across both classes:
  (10) $$\mathrm{Accuracy}=\frac{TP+TN}{TP+TN+FP+FN}$$
- Recall (Sensitivity)—evaluates the classifier’s ability to identify true positive instances:
  (11) $$\mathrm{Recall}=\frac{TP}{TP+FN}$$
- Precision (Positive Predictive Value)—represents the proportion of predicted positives that are actually positive:
  (12) $$\mathrm{Precision}=\frac{TP}{TP+FP}$$
- F1-Score—the harmonic mean of precision and recall, providing a balanced assessment:
  (13) $$F1=2\cdot \frac{\mathrm{Precision}\cdot \mathrm{Recall}}{\mathrm{Precision}+\mathrm{Recall}}$$

These indicators are critical in medical domains, such as preeclampsia screening, where false negatives can result in undetected high-risk cases, and false positives may lead to unnecessary interventions [53].

In addition, the Receiver Operating Characteristic (ROC) curve and Area Under the Curve (AUC) were calculated to provide threshold-independent evaluation. The ROC plots the true positive rate (TPR) against the false positive rate (FPR), defined as

(14) $$TPR=\frac{TP}{TP+FN},\;FPR=\frac{FP}{FP+TN}$$

An important consideration is the K-fold term. K-fold cross-validation is a technique that splits data into *k* subsets (folds), trains the model on k−1 folds, and tests it on the remaining fold, repeating this process *k* times to evaluate performance.

The AUC summarizes classifier performance. The points on the ROC curve depend on the number of K-folds applied, TPR on the y-axis and the FPR on the x-axis.

Finally, all models were validated using stratified *k*-fold cross-validation, ensuring balanced class distribution in each fold. This technique minimizes bias and variance in performance estimates and is widely adopted in machine learning for model generalization evaluation [54].

#### 3.5.2. Stage 5.2: Model Comparison

To validate the effectiveness of the SK-MOEFS classifier, a comparative evaluation was conducted using two well-known interpretable models: DT and RF. These baseline models were implemented using the MLJAR platform [55], which applies metaheuristics to optimize model configurations.

DT and RF are widely recognized in machine learning for their simplicity, performance, and interpretability in various clinical prediction tasks [56,57]. The DT model recursively partitions the feature space by maximizing the *IG*, defined as

(15) $$IG\left(D,A\right)=Entropy\left(D\right)-\sum_{v\in Values(A)}\frac{|D_{v}|}{\left|D\right|}\cdot Entropy\left(D_{v}\right)$$

where the entropy of a dataset *D* is given by

(16) $$Entropy\left(D\right)=-\sum_{i=1}^{c}p_{i}\cdot \log_{2}\left(p_{i}\right)$$

with $p_{i}$ representing the proportion of instances belonging to class *i*, and *c* the total number of classes.

Alternatively, the Gini index can be used as a splitting criterion, defined as

(17) $$Gini\left(D\right)=1-\sum_{i=1}^{c}p_{i}^{2}$$

RF extends DT by building an ensemble of *B* trees, each trained on a different bootstrap sample and using a random subset of features. Predictions are made via majority voting:

(18) $$\hat{y}=\mathrm{mode}\left(\left\{T_{1}\left(x\right),T_{2}\left(x\right),...,T_{B}\left(x\right)\right\}\right)$$

This ensemble approach improves accuracy and robustness, reducing the risk of overfitting compared to a single decision tree [58].

All models were evaluated under identical conditions: a 75/25 stratified train–test split and 4-fold cross-validation, ensuring balanced class representation across folds.

### 3.6. Stage 6: Model Interpretation

The interpretability of the SK-MOEFS model is essential for clinical use as it enables transparent and traceable decision-making. This stage involves two key components: aggregation and defuzzification. When multiple fuzzy rules are activated, their outputs are aggregated using a t-norm operator (e.g., minimum), forming a global fuzzy inference.

**Figure 3:**
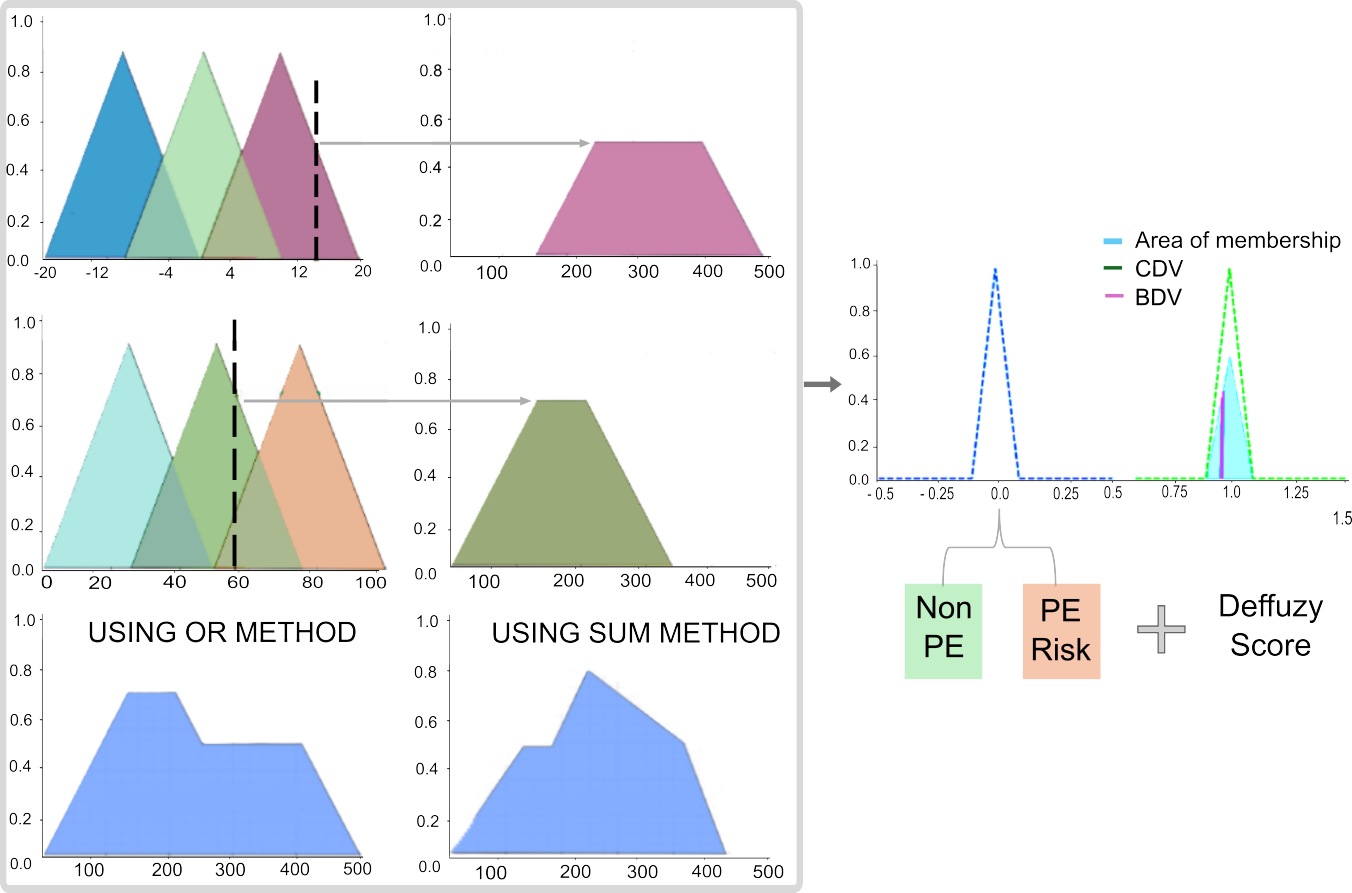
Defuzzification model: On On the left, the rule aggregation stage selects the activated rules and combines their contributions using aggregation operators (e.g., OR, SUM). On the right, the defuzzification stage shows the resulting fuzzy output for PE risk, including the area of membership and the application of the bisector (BDV) and centroid (CDV) methods to obtain the final crisp score. Color and arrow annotations indicate the propagation of activation through the fuzzy logic pipeline.

To obtain a crisp output, defuzzification methods are applied. The model incorporates two classical strategies [59]: (i) the centroid method, which computes the center of gravity of the output set, and (ii) the bisector method, which divides the fuzzy area into two equal parts. These methods provide interpretable continuous-valued predictions (Figure 3).

The fuzzy reasoning module is implemented based on Georgiev’s open-source system [60], ensuring reproducibility. This rule-based interpretability aligns with ethical principles for explainable AI in healthcare [61,62], offering clinicians not only predictive accuracy but also insight into the reasoning behind each decision.

## 4. Results

This chapter presents a structured and in-depth evaluation of the SK-MOEFS framework for the classification of preeclampsia, highlighting its predictive performance, interpretability, and clinical relevance. The model’s behavior is analyzed across multiple phases, including parameter optimization, rule extraction, fuzzy inference, and validation on unseen balanced clinical data. The results demonstrate that SK-MOEFS achieves high recall, strong generalization capacity, and transparent decision-making grounded in linguistically meaningful fuzzy rules. Compared to conventional models, its native explainability and cautious inference approach make it particularly suited for clinical decision-making, where interpretability and assisted diagnostic reliability are critical.

The chapter begins with the identification of optimal model parameters through a multi-criteria selection strategy aimed at maximizing minority-class sensitivity—detailed in the following section.

### 4.1. Optimal Parameter Configuration of the SK-MOEFS Model

To ensure both high classification performance and clinical interpretability, the SK-MOEFS model was tuned through a rigorous parameter optimization strategy. Among the multiple evaluation criteria, the use of normalized differences between true and false classifications emerged as a central step in selecting the best-performing configurations. This approach emphasized sensitivity to the minority class—preeclampsia—reflecting the clinical importance of minimizing false negatives in maternal risk scenarios.

**Figure 4:**
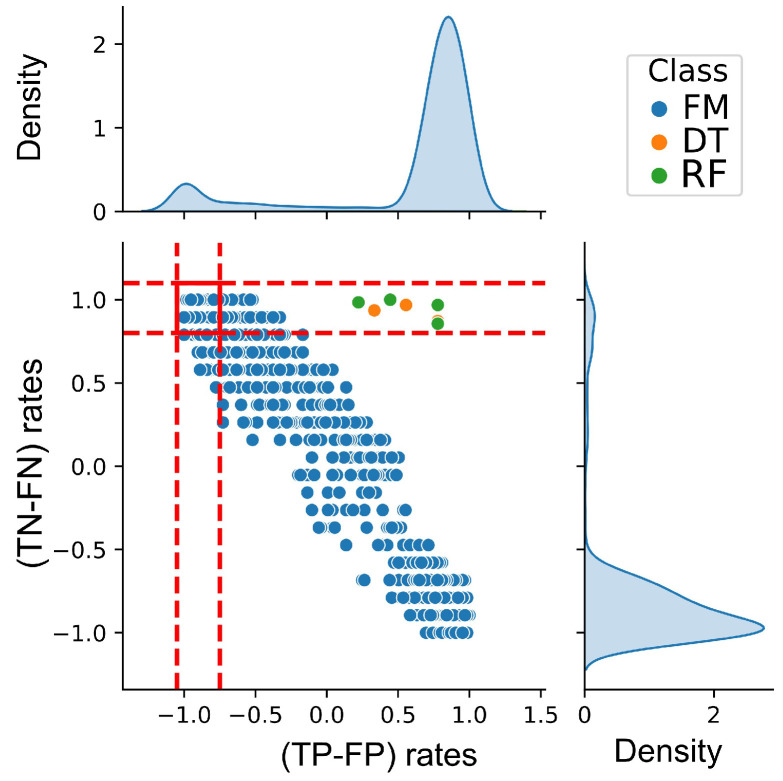
Joinplot showing normalized difference rates of TN–FN and TP–FP for selecting configurations of the models. FM is fuzzy model, including point results from parameter configurations. DT is decision tree. RF is random forest. Red lines indicate the intersection zone where normalized differences are maximum.

As illustrated in Figure 4, a comparative assessment of model configurations was conducted to determine their effectiveness in distinguishing between positive and negative classes. Each point in the plot represents a specific configuration of the FM, DT, or RF models, which were evaluated based on the normalized differences between true positives and false positives (TP–FP), as well as true negatives and false negatives (TN–FN). The red horizontal and vertical lines in the plot signify a critical region that acts as a visual threshold; configurations located within this area indicate an enhanced ability to discriminate the minority class. In the initial fuzzy model (FM) configurations, characterized by over 70 rules prior to reduction, most points fell outside the red-bounded region. These configurations demonstrated inadequate separation between correct and incorrect classifications and performed suboptimally compared to the DT and RF models in terms of recall. This is evidenced by their low normalized difference values, which reflect limited discriminative power. Configurations residing within the intersection zone were prioritized as they demonstrated an ability to address the inherent dataset imbalance commonly found in real-world clinical data, thereby realigning the model’s bias toward clinically relevant classifications.

The normalization used was defined as follows:

(19) $$\mathrm{TP}-\mathrm{FP}\;\mathrm{rates}=\frac{TP-FP}{TP+FP},\;\mathrm{TN}-\mathrm{FN}\;\mathrm{rates}=\frac{TN-FN}{TN+FN}$$

This scale-independent representation highlights differences in class-specific behavior that are not evident when looking at overall accuracy alone. This distinction is especially crucial in preeclampsia detection, where minimizing false negatives is essential. Finally, the model starts with configurations located at the red threshold limits, corresponding to models with over 70 rules. These initial configurations show better overall class balance but struggle to discriminate the minority class. After optimization, reducing the number of rules to six enhances the model’s ability to capture minority patterns and improves class separation.

Based on this rationale, the selected parameter set was as follows:

max_depth = 15, discr_minImpurity = 0.005, discr_minGain = 0.02, minGain = 0.001, minNumExamples = 4, max_prop = 1.0, and discr_threshold = 2.

Moreover, although initial configurations assumed five membership functions (MFs) per variable, further evaluation revealed that a model with four functions offered a better balance between performance consistency and rule simplicity. As summarized in Table 7, this configuration retained high accuracy (93%) while minimizing ruleset complexity, which enhances transparency and applicability in clinical environments.

Although the six-MF model slightly improved recall (0.74), it also increased complexity, making it less desirable in a real-world clinical environment where fewer more interpretable rules are preferred. Consequently, the four-MF configuration was selected as the most clinically viable alternative.

The final model adopted a configuration with four fuzzy membership functions and reduced the ruleset from eighty-two to six rules. This compact rule base aligns with the framework’s core objective: to enable interpretable, clinically aligned AI support systems that do not compromise diagnostic rigor. By prioritizing recall during optimization, the model remains focused on capturing at-risk individuals, a critical factor for effective early intervention in preeclampsia cases.

In summary, this optimization strategy reinforces the clinical applicability of the explainable fuzzy framework. By guiding parameter selection through visual and performance-based analysis, it supports the development of decision-support tools that are not only accurate but also aligned with clinical reasoning and risk tolerance standards.

### 4.2. Performance Classification of the Implemented Models

To evaluate the classification capacity of the proposed fuzzy model (FM), we conducted a comparative analysis with two commonly used XAI models, DT and RF. The evaluation was based on multiple metrics, including accuracy, recall, precision, F1-score, error rate, and the AUC, using a 75/25 train–test split stratified and 4-fold stratified cross-validation.

A key methodological consideration was ensuring the distributional consistency of the imputed data. Since part of the dataset was completed through imputation methods (as described in Section 3.3), we verified that the imputed values were equally represented in both the training and testing sets by applying stratification during partitioning. This approach guaranteed that imputation would not become a confounding factor affecting model performance or generalizability.

As shown in Table 8, all three models achieved strong classification results, with accuracies exceeding 90%. The DT model reported the highest accuracy (94%) and F1-score (0.79), reflecting a well-balanced prediction capability. The RF model achieved the highest AUC (0.95), indicating optimal global discriminative power. However, the FM demonstrated the highest recall (0.88), highlighting its effectiveness in identifying true preeclampsia cases.

While the FM did not outperform DT and RF in terms of overall precision or accuracy, its superior recall supports its clinical relevance—ensuring that high-risk patients are more reliably detected. This aligns with the framework’s design philosophy, which prioritizes sensitivity to critical medical outcomes over marginal improvements in aggregate metrics.

**Figure 5:**
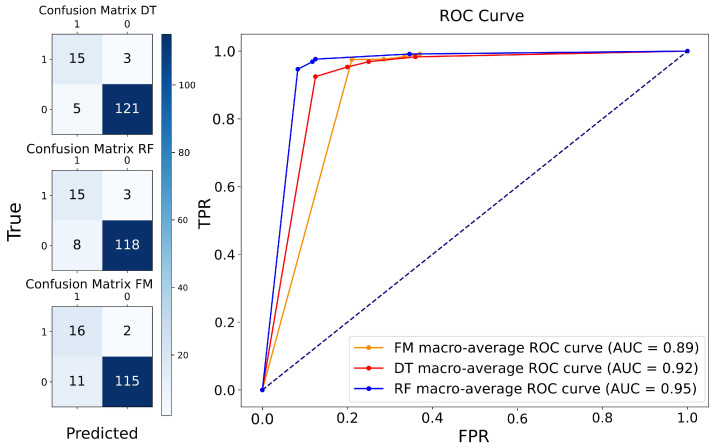
Discriminatory properties of the models during classification. First column: confusion matrix results for three models; second column: ROC curves of three models compared (4 K-folds). FM: fuzzy model; DT: decision tree; RF: random forest. The dotted diagonal line in the ROC plot represents the performance of a random classifier (AUC = 0.5), and serves as a baseline for evaluating model performance.

The ROC curves in Figure 5 (right column) further illustrate the models’ discriminative properties. While RF maintains the best AUC, the FM shows a consistently favorable trade-off between sensitivity and specificity. Meanwhile, the confusion matrices (Figure 5, left column) confirm that the fuzzy model sustains a balanced performance despite dataset imbalance.

In all the cases, a fixed random seed (123) was applied to ensure reproducibility across runs. Together with stratification, this reinforced the reliability of the comparisons and reduced the influence of sampling variance or imputation bias.

The fuzzy model demonstrates competitive performance in detecting preeclampsia cases. Its slightly lower overall accuracy is offset by a pronounced gain in recall, supporting its implementation in clinical scenarios where identifying true positives is imperative.

### 4.3. SK-MOEFS’s Rules

One of the most distinctive strengths of the proposed explainable fuzzy framework is its ability to generate a compact and interpretable set of fuzzy rules that articulate the model’s reasoning process. These rules serve as the core mechanism for translating abstract data relationships into semantically meaningful statements that can be directly interpreted by clinicians, a critical factor in clinical decision support systems.

After model training and optimization, the system initially produced 76 fuzzy rules. Through multi-objective evolutionary reduction, this number was refined to a final set of six rules that preserved classification performance while significantly improving interpretability. Each rule includes antecedents based on fuzzy linguistic variables—such as MAP, BMI, and maternal age—and a consequent indicating the probability of class membership (i.e., preeclampsia or no-preeclampsia). The reduced ruleset is listed below:

Rule 1: If MAP is VL and BMI is H, then p is 0[0.836 0.164].
Rule 2: If MAP is L and BMI is L, then p is 0[1. 0.].
Rule 3: If MAP is L, then p is 0[9.993e-01 7.198e-04].
Rule 4: If MAP is H, then p is 1[0.480 0.520].
Rule 5: If MAP is H and BMI is VL  and age is L, then p is 0[0.7920 0.208].
Rule 6: If MAP is H and BMI is M  and age is L, then p is 1[0.335 0.664].

These rules are hierarchically structured and reflect the fuzzy decision tree logic underlying the FM. Their interpretability stems from the use of human-understandable linguistic descriptors (e.g., “high BMI” or “low MAP”) that correspond to clinical variables routinely collected during prenatal care. Each rule also provides a class probability vector, indicating the model’s confidence in the prediction. This probabilistic output enhances transparency by allowing clinicians to evaluate the strength of each classification. For example: Rule 4 reflects an intuitive and clinically plausible insight: higher blood pressure (MAP) is more strongly associated with preeclampsia risk. Rules 5 and 6 further integrate multiple variables, suggesting interactions that modulate risk—such as age and BMI in conjunction with MAP—which mirrors how risk factors are evaluated in obstetric practice.

**Figure 6:**
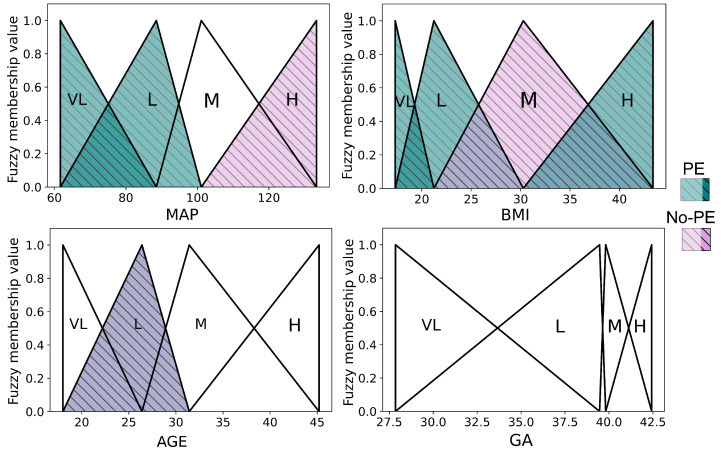
Fuzzy membership functions of SK-MOEFS for classification of preeclampsia. Gestational age (GA) variable was not activated. Colored areas represent the regions activated by each class: blue-green tones with diagonal lines indicate samples associated with the PE group (Preeclampsia), and purple-pink tones indicate the No-PE group (non-preeclampsia). These overlays illustrate class-specific activation within each fuzzy set.

Figure 6 presents fuzzy membership functions used to model clinical variables within a fuzzy-logic-based system. Each subplot shows how the values of a continuous variable—MAP (mean arterial pressure), BMI (body mass index), age (maternal age), and GA (gestational age)—are grouped into linguistic categories such as very low (VL), low (L), medium (M), and high (H) using triangular functions. The groups of colored triangles refer to two distinct populations: the red triangles represent the preeclampsia (PE) group, while the green triangles represent the non-preeclampsia (No-PE) group. These functions illustrate how each variable is interpreted differently between the groups, capturing variations in clinical patterns between PE and No-PE patients.

### 4.4. SK-MOEFS’s Translation and Defuzzification

**Figure 7:**
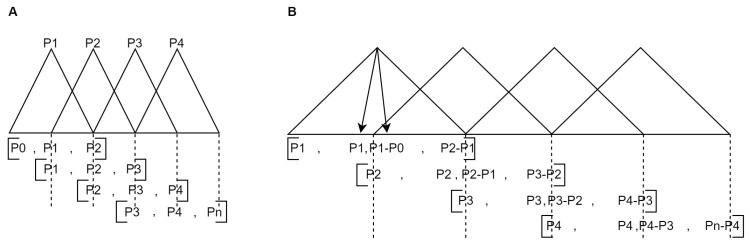
Translation model. (**A**). Triangular fuzzy model from triangular partitions. (**B**). Trapezoidal fuzzy model from triangular partitions.

The SK-MOEF system efficiently generates fuzzy rules that aid in understanding the criteria for making predictions. However, a defuzzification process is required for a more detailed analysis of a specific case. Given specific values, this process will generate a patient’s membership level to a particular class. SK-MOEF systems build the triangular fuzzy set by adding anchor arrays, constructed appending the first and last elements of the partition to itself. Then, the anchor arrays build each triangle defined by a triangular membership function, with a peak at 1.0 and the other element at 0.0, as shown in Figure 7A. Finally, we transform the triangles to the scikit-fuzzy trapezoid format (Table 9). The first value of the triangle is repeated to establish the start and maximum of the partition. The next two values are the differences between the anchors of each triangle, as shown in Figure 7B.

The result for the case study example yields the following results:

It is important to note that the obtained fuzzy partitions correspond only to the activated variables. Thus, out of the five initially considered variables, only three were activated: MAP, BMI, and age. Therefore, it can be inferred that these three variables contribute to predicting the studied class (preeclampsia).

**Figure 8:**
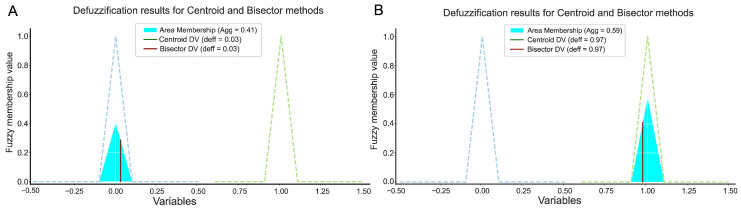
Results of the defuzzification for two specific cases. In (**A**), the outcome of a patient with PE incorrectly classified (false negative) is shown. In (**B**), the outcome of a patient with PE correctly classified (true positive) is displayed.

In Figure 8, the left-hand side shows the fuzzy rule activation for each input variable and how the system resolves their contributions. For case **A**, although some activation occurred in rules associated with elevated MAP, the combined influence was not sufficient to cross the decision threshold toward class 1. The final defuzzified output was 0.41, leading to a negative classification.

In contrast, case **B** activated rules with higher degrees of membership in the high-risk region. Specifically, fuzzy terms such as “high MAP”, “medium BMI”, and “low age” triggered a strong response in Rule 6, which is associated with a higher probability of preeclampsia. The aggregated result yielded a crisp value of 0.59, sufficient to classify the instance as PE (class 1). This illustrates how the fuzzy system accumulates partial evidence from multiple rules to reach a probabilistic decision.

While the use of linguistic variables may seem to simplify continuous data, the fuzzy decision tree construction based on fuzzy information gain inherently accounts for multivariate interactions. For example, Rules 5 and 6 illustrate how combinations of MAP, BMI, and age contribute to nuanced decision-making. Thus, the model captures interaction effects without requiring opaque or uninterpretable structures.

### 4.5. Explainability of the Machine Learning Models

We compare the explainability of the FM with conventional interpretable models such as DT and RF, with a focus on clinical applicability. While DT and RF rely on post hoc techniques such as SHAPs to infer variable importance, the FM incorporates explainability as a native feature through the use of explicit fuzzy rules and clinically relevant variables.

These fuzzy rules operate on input variables that are mapped to linguistic terms. This enables the construction of clear interpretable decision paths, allowing healthcare professionals to trace predictions back to rule-based logic.

**Figure 9:**
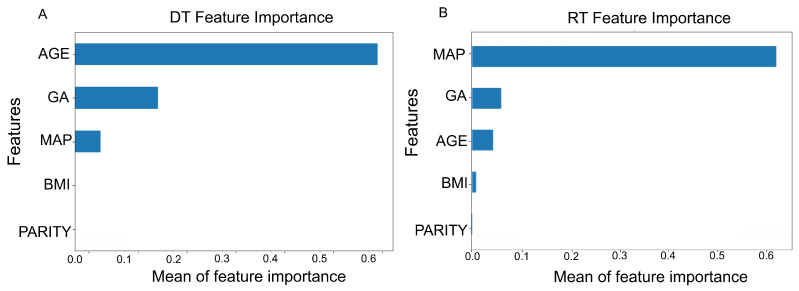
Feature importance of DT and RF models. Subfigure (**A**) shows the mean feature importance values for the Decision Tree (DT), while subfigure (**B**) presents the corresponding values for the Random Forest (RF). AGE was the most influential variable for the Decision Tree, followed by MAP and GA. In the Random Forest model, MAP was the most influential variable, followed by GA and AGE. The bars represent the normalized importance of each feature, with higher bars indicating greater influence on the model’s decision-making process.

By contrast, SHAP values quantify feature contributions but do not provide an explicit reasoning process, and their interpretation often requires technical expertise. While SHAP-based insights are valuable, they may lack the semantic clarity and direct clinical relevance offered by rule-based systems (Figure 9).

In the DT in Figure 9A, AGE stands out as the most important feature, followed by GA and MAP. Other variables like BMI and PARITY contribute little to the model, with PARITY showing virtually no importance.

In contrast, the RF in Figure 9B highlights MAP as the dominant feature, far outweighing the rest in importance. GA and AGE follow but with significantly lower mean SHAP values, and BMI and PARITY are again almost negligible in influence.

This differs from the findings of the SK-MOEF system, where GA is inactive and BMI is active. While these results guide prediction, the analysis is less comprehensive than the fuzzy model’s.

### 4.6. Validation Case

This section presents an external evaluation of the FM model using an independent validation dataset, designed to simulate real-world clinical deployment scenarios. Unlike the training set, this new dataset featured a more balanced class distribution, enabling a more realistic assessment of model behavior in clinical conditions.

All three models, FM, DT, and RF, achieved outstanding performance, with overall accuracy above 98% and high scores across the recall, F1 and error (Table 10). These results indicate that the fuzzy model retains strong generalization capability, suggesting that its learned rules are not overfitted to the training data.

However, the FM model failed to produce predictions for 10 cases due to the absence of activated rules for those input combinations. While this reflects the model’s conservative inference strategy—favoring confident decision-making—it also reveals a limitation in rule coverage that must be addressed to ensure full applicability in diverse patient populations.

Despite this limitation, all the predictions made by the fuzzy model were correct, further supporting its diagnostic reliability and value as an interpretable decision-support tool in clinically sensitive domains such as preeclampsia detection.

## 5. Discussion

This study introduces an innovative fuzzy-logic-based approach (SK-MOEFS) designed to improve the explainability of machine learning (ML) models applied to the classification of hypertensive disorders, particularly preeclampsia. By integrating multi-objective evolutionary algorithms with fuzzy inference systems, the framework achieves a balanced trade-off between model performance and interpretability—two critical features for clinical decision support.

The implementation of a fuzzy rule translation model and defuzzification mechanism provides an interpretable structure for medical professionals, enabling clear traceability from input variables to clinical recommendations. This feature enhances transparency and aligns closely with the principles of explainable artificial intelligence (XAI) increasingly demanded in medical AI applications.

Missing and inconsistent data are effectively managed through the application of Bayesian PCA (BPCA), a well-regarded imputation strategy selected after an extensive evaluation of 18 algorithms. This method was chosen for its ability to preserve clinical plausibility and maintain the integrity of data distributions. Furthermore, the fuzzy logic system provides intrinsic robustness against data imprecision and noise thanks to its formulation of linguistic rules. Collectively, these qualities make the model exceptionally well-suited for implementation in real-world clinical environments, where data quality may be compromised by systemic or operational inconsistencies.

Our study presents a fuzzy-logic-based method that allows for a detailed analysis of explainability. As mentioned earlier, our approach not only generates fuzzy rules for decision-making but also facilitates the identification of activated variables during classification. This capability is crucial for understanding which information significantly contributes to classification, providing detailed insights into the model’s inner workings. Most works proposing an explainable model do not specifically address explainability [63].

In terms of performance, the explainable fuzzy framework achieved an accuracy of 91% and an AUC of 0.89, outperforming earlier interpretable models such as those proposed by Zhang et al. [8] (AUC = 0.84) and Liu et al. [7] (AUC = 0.86) while also demonstrating improved precision over the system introduced by Maric et al. [5] (AUC = 0.79). However, it is acknowledged that higher AUC values were achieved in models using advanced black-box techniques, such as the XGBoost-based model by Jhee et al. [6] (AUC = 0.92) and ensemble strategies in Sufriyana et al. [64] (AUC = 0.93).

Despite slightly lower performance metrics in comparison to these black-box approaches, the explainable fuzzy framework offers native interpretability, avoiding reliance on post hoc tools like SHAPs or LIMEs. While models like those of Espinilla et al. [19] and Pham et al. [22] incorporated explainable elements through fuzzy logic and fuzzy knowledge graphs, these often introduced a large number of rules or lacked full transparency in decision logic. In contrast, SK-MOEFS reduced its rule base to just six linguistically coherent rules, improving both usability and interpretability without sacrificing diagnostic relevance.

An important aspect is that the fuzzy model achieved the best recall value. This implies that the method is focused on favoring sensitivity, a critical metric in medicine that ensures efficient detection of positive cases [61]. This approach reduces errors in identifying sick individuals as healthy at the expense of other metrics.

Furthermore, the model’s behavior was tested against an unseen more balanced validation dataset. All three evaluated models, FM, DT, and RF, achieved excellent generalization, with accuracies above 98%. However, only the fuzzy system showed prediction abstention in 10 cases due to unactivated rule combinations. While this cautious behavior may be seen as a limitation in terms of coverage, it also reflects a risk-averse design, which can be valuable in clinical scenarios where erroneous predictions carry high stakes.

Another key contribution of this work is its alignment with clinical reasoning. By mapping continuous input variables (e.g., MAP, BMI, and age) into fuzzy linguistic terms, the system enables physicians to understand how patient characteristics relate to risk, fostering greater trust and clinical integration. This stands in contrast to neural-network-based solutions, which—despite strong performance—often lack sufficient transparency for direct clinical interpretation.

It is important to highlight that the FM model has already demonstrated robust generalization capabilities. It was trained on a merged dataset composed of four distinct clinical sources from geographically and demographically diverse populations, including cohorts from England, India, Thailand, and Vietnam. This diversity strengthens the model’s representativeness and supports its applicability in broader real-world settings, as evidenced by the performance consistency observed in its external validation.

The explainable fuzzy framework successfully combines robustness and interpretability. It demonstrates strong predictive power relative to the existing interpretable models while offering a degree of transparency that is still largely unmatched in most AI-based approaches to preeclampsia classification. This makes it a strong candidate for integration into clinician-facing tools. Future efforts should focus on enhancing rule coverage, adaptive learning for evolving clinical data, and real-time integration with electronic health records.

## 6. Conclusions

This research presents an interpretable fuzzy-based classification explainable fuzzy framework for identifying preeclampsia during the third trimester of pregnancy across multi-ethnic populations. The proposed system demonstrates robust predictive capability, achieving up to 91% accuracy and a recall of 88%, prioritizing sensitivity over overall accuracy to minimize the risk of false negatives—a critical objective in obstetric clinical practice.

A key innovation of this approach lies in its integration of fuzzy rule generation with multi-objective evolutionary optimization, allowing for the automatic derivation of concise and interpretable rules that preserve diagnostic reliability. Compared to high-performance black-box models such as XGBoost (AUC≈0.92) or ensemble trees (AUC≈0.93), as reported by Jhee et al. and Sufriyana et al., SK-MOEFS offers comparable discrimination power while providing native transparency, avoiding the need for post hoc interpretability methods like SHAPs or LIMEs.

The implementation of linguistically interpretable rules, supported by defuzzification mechanisms, allows clinicians to understand not only the model’s decisions but also the contributing factors and their degrees of membership. This design enhances user trust and aligns with contemporary ethical standards for responsible AI in healthcare.

Our method enhances user confidence by enhancing the transparency and understanding of the model’s decision-making process. It aligns with the latest trends in ethics and accountability in AI system development [62]. This integration of ethical principles strengthens our proposal’s practical and sustainable application in diverse contexts.

The framework’s generalizability was further supported by a validation phase on an independent balanced dataset that includes three ethnic populations (England, India, and Thailand). While the system correctly classified most instances, it abstained from decision-making in a small fraction of cases where no rule activation occurred. Rather than a limitation, this behavior highlights the model’s conservative inference policy, ensuring that decisions are only made when rule support is sufficient. The final set of six fuzzy rules were not arbitrarily chosen. This concise ruleset retains the most relevant and statistically discriminative patterns, ensuring interpretability while maintaining classification performance. By providing clinicians with a clear probabilistic explanation of predictions, the model supports decision-making in complex clinical scenarios where understanding the underlying logic is as important as the accuracy of the prediction itself.

Moreover, the multi-ethnic dataset employed—although limited to five common features—represents a foundational effort toward dataset normalization in preeclampsia research. The encouraging validation results support the inclusion of this dataset in future benchmarking studies and model generalization assessments.

This research contributes to the broader goal of developing explainable and clinically trustworthy AI systems. The explainable fuzzy framework effectively bridges data-driven modeling with human-understandable logic, reinforcing its suitability for integration into decision-support environments in maternal care.

Future work will focus on extending rule coverage and incorporating adaptive learning techniques to handle evolving clinical data patterns over time. Although the current guidelines provide valuable insights, they may not yet capture all the patient-specific variations, which could limit the model’s scalability and long-term applicability. Addressing these challenges is essential for ensuring the robustness and clinical relevance of the proposed system across diverse populations and settings.

## Figures and Tables

**Table 1:** Descriptive analysis of the datasets used in this research.

Reference	Total (n)	No-PE/PE	Variable	Mean ± Std	Min–Max
Petry et al. [42]	438	431/7	MAP	84.95±9.58	61–133.33
			BMI	23.87±4.22	16.61–43.40
			Age	33.40±4.28	21.2–45.2
			Parity	1.71±0.84	1–6
			GA	40.18±1.23	27.86–42.43
Tatapudi et al. [43]	100	69/31	MAP	102.68±16.76	73–140
			Age	23.73±3.35	18–32
			Parity	1.24±0.43	1–2
Thitivichienlert [44]	34	0/34	MAP	111.22±7.60	99.33–132.66
			BMI	26.20±4.82	17.31–38.22
			Age	29.08±6.88	18–41
			Parity	1.61±0.49	1–2
			GA	37.20±2.33	30–41
Pham [22]	198	111/85	MAP	98.75±20.40	50–163.33
			BMI	24.30±2.56	18.67–34.06
			Age	30.91±2.56	18.67–34.06
			Parity	1.72±0.92	0–5
			GA	32.03±2.35	21–35

**Table 2:** Missing value datasets.

Reference	Total	MAP	BMI	Age	Parity	GA
Petry et al. [42]	438	0	38	13	0	2
Thitivichienlert [44]	34	0	4	0	0	0

**Table 3:** Descriptive analysis of imputed data for Tatapudi and Pasumarthy [43] dataset.

Dataset	Variable	Mean ± Std	Min–Max
Total	BMI	24.81±1.12	23.10–26.91
	GA	39.57±0.75	38.70–40.41
PE	BMI	25.98±0.31	26.23–26.91
	GA	38.82±0.05	38.70–38.94
No-PE	BMI	24.28±0.91	23.10–26.21
	GA	39.91±0.68	38.77–40.41

**Table 4:** Summary of combined dataset including imputed data.

Dataset	Total (n)	Variable	Mean ± Std	Min–Max
Combined	574	MAP	89.59±14.01	61.66–140
		BMI	24.16±3.77	16.61–43.40
		Age	31.44±5.65	18–45
		Parity	1.62±0.79	1–6
		GA	39.91±1.44	27.86–42.42
No PE	502	MAP	86.21±18.70	61.66–133.33
		BMI	23.86±3.72	16.61–43.40
		Age	32.06±5.27	18–45
		Parity	1.64±0.82	1–6
		GA	40.16±1.17	27.86–42.42
PE	72	MAP	113.18±11.48	72.66–140
		BMI	26.25±3.52	17.31–38.36
		Age	27.01±6.63	18–43
		Parity	1.51±0.55	1–3
		GA	38.13±1.89	30–41.57

**Table 5:** Grid parameters evaluated in the machine learning models: max_depth: maximum depth of fuzzy decision tree; discr_minImpurity: minimum impurity for a fuzzy set; discr_minGain: minimum entropy gain; discr_threshold: if discr_threshold ≠ 0, the discretization is stopped at n = discr_threshold + 2 fuzzy sets; minGain: minimum entropy gain for a split; minNumExample: minimum number of examples for a node; max_prop: minimum proportion of samples pertaining to the same class in the same node for stop splitting.

Parameter	Values
max_depth	[5, 10, 15]
discr_minImpurity	[0.001, 0.005, 0.01, 0.02, 0.05, 0.1]
discr_minGain	[0.001, 0.005, 0.01, 0.02, 0.05, 0.1]
minGain	[0.001, 0.005, 0.01, 0.02, 0.05, 0.1]
minNumExamples	[1, 2, 3, 4]
max_prop	1.0
discr_threshold	[0, 1, 2, 3]
MF	5

**Table 6:** Confusion  matrix.

	Predicted	Predicted	
	Positive	Negative	
Actual	TP	FN	Recall
Positive	*True Positive*	*False Negative*	$\frac{TP}{TP+FN}$
Actual	FP	TN	Specificity
Negative	*False Positive*	*True Negative*	$\frac{TN}{TN+FP}$
	Precision	Predictive Value	Accuracy
	$\frac{TP}{TP+FP}$	$\frac{TN}{TN+FN}$	$\frac{TP+TN}{TP+TN+FN+FP}$

**Table 7:** Best metric results for optimizing the membership functions of SK-MOEFS.

MF	4	5	6
Number of Rules	82	118	117
Error	0.07	0.07	0.07
Accuracy	0.93	0.93	0.93
F1-Score	0.71	0.71	0.74
Precision	0.80	0.80	0.74
Recall	0.63	0.63	0.74

**Table 8:** Performance metrics of the proposed model.

Measure	Decision Tree	Random Forest	Fuzzy Model
Accuracy	0.94	0.92	0.91
Precision	0.75	0.65	0.60
Recall	0.83	0.83	0.88
F1-Score	0.79	0.73	0.71
Error	0.055	0.076	0.090
AUC	0.92	0.95	0.89

**Table 9:** MAP, BMI, and age partition in trapezoid format.

	MAP	BMI	AGE
VL	[61.67, 61.67, 0.0, 26.84]	[17.31, 17.31, 0.0, 3.92]	[18.0, 18.0, 0.0, 8.40]
L	[88.51, 88.51, 26.84, 12.58]	[21.23, 21.23, 3.92, 9.05]	[26.4, 26.4, 8.40, 5.02]
M	[101.09, 101.09, 12.58, 32.24]	[30.27, 30.27, 9.05, 13.12]	[31.42, 31.42, 5.02, 13.78]
H	[133.33, 133.33, 32.24, 0.0]	[43.40, 43.40, 13.12, 0.0]	[45.2, 45.2, 13.78, 0.0]

**Table 10:** Performance metric models.

Measure	Decision Tree	Random Forest	Fuzzy Model
Accuracy	0.98	1.0	0.98
Precision	0.96	1.0	1.0
Recall	1.0	1.0	0.96
F1-Score	0.98	1.0	0.98
Error	0.015	0.0	0.015
Confusion Matrix	$\left[\begin{array}{cc} 85 & 0\\ 3 & 111 \end{array}\right]$	$\left[\begin{array}{cc} 85 & 0\\ 0 & 114 \end{array}\right]$	$\left[\begin{array}{cc} 73 & 3\\ 0 & 113 \end{array}\right]$

## Data Availability

The data used in this study can be obtained from their original repositories: https://www.repository.cam.ac.uk/items/2e890286-663c-4f38-af71-cb972ce455b9 (accessed on 15 April 2025), https://data.mendeley.com/datasets/d72zr4xggx/1 (accessed on 15 April 2025), https://data.mendeley.com/datasets/ffpx6t6ky4/1 (accessed on 15 April 2025), https://github.com/FKGHUST/Preeclampsia (accessed on 15 April 2025).

## Abbreviations

The following abbreviations are used in this manuscript:

AI	Artificial Intelligence
SK-MOEFS	Sparse Kernel-Based Multi-Objective Evolutionary Fuzzy System
XAI	Explainable Artificial Intelligence
FRBS	Fuzzy-Rule-Based System
MOEA	Multi-Objective Evolutionary Algorithm
ML	Machine Learning
GFS	Genetic Fuzzy System
RB	Rule-Based
FRBC	Fuzzy-Rule-Based Classifier
TRL	Total Rule Length
MOEL	Multi-Objective Learning Scheme
MAP	Mean Arterial Pressure
BMI	Body Mass Index
GA	Gestational Age
